# Cognitive decline according to amyloid uptake in patients with poststroke cognitive impairment

**DOI:** 10.1097/MD.0000000000027252

**Published:** 2021-09-24

**Authors:** Bora Yoon, Dong Won Yang, Yun-Jeong Hong, Taewon Kim, Seunghee Na, Sang-Mi Noh, Hye Lim Park, Bon D. Ku, Young Soon Yang, Hojin Choi, Jae-Won Jang, Seongheon Kim, Yerim Kim, YongSoo Shim

**Affiliations:** a Department of Neurology, Konyang University Hospital, Konyang University College of Medicine, Daejeon, Republic of Korea; b Department of Neurology, The Catholic University of Korea Seoul St. Mary's Hospital, Seoul, Republic of Korea; c Department of Neurology, The Catholic University of Korea Uijeongbu St. Mary's Hospital, Uijeongbu, Republic of Korea; d Department of Neurology, The Catholic University of Korea Incheon St. Mary's Hospital, Incheon, Republic of Korea; e Department of Neurology, The Catholic University of Korea St. Vincent's Hospital, Suwon, Republic of Korea; f Division of Radiology, Department of Nuclear Medicine, The Catholic University of Korea Eunpyeong St. Mary's Hospital, Seoul, Republic of Korea; g Department of Neurology, International St. Mary's Hospital, Catholic Kwandong University College of Medicine, Incheon, Republic of Korea; h Department of Neurology, Soonchunhyang University College of Medicine, Cheonan Hospital, Cheonan, Republic of Korea; i Department of Neurology, Hanyang University Guri Hospital, Guri, Republic of Korea; j Department of Neurology, Kangwon National University Hospital, Kangwon National University College of Medicine, Chuncheon, Republic of Korea; k Department of Neurology, Kangdong Sacred Heart Hospital, Hallym University College of Medicine, Seoul, Republic of Korea; l Department of Neurology, The Catholic University of Korea Eunpyeong St. Mary's Hospital, Seoul, Republic of Korea.

**Keywords:** amyloid, cerebral infarct, clinical trial, cognitive impairment, prognosis

## Abstract

**Background and purpose:**

Poststroke cognitive impairment (PSCI) is common, but the impact of β-amyloid (Aβ) on PSCI is uncertain. The proposed study will investigate amyloid pathology in participants with PSCI and how differently their cognition progress according to the amyloid pathology.

**Methods:**

This multicenter study was designed to be prospective and observational based on a projected cohort size of 196 participants with either newly developed cognitive impairment, or rapidly aggravated CI, within 3 months after acute cerebral infarction. They will undergo ^18^F-flutemetamol positron emission tomography at baseline and will be categorized as either amyloid-positive (A+) or amyloid-negative (A−) by visual rating. The primary outcome measures will be based on Korean Mini-Mental State Examination changes (baseline to 12 months) between the A+ and A− groups. The secondary outcome measures will be the dementia-conversion rate and changes in the Korean version of the Montreal Cognitive Assessment (baseline to 12 months) between the A+ and A− groups.

**Conclusions:**

This study will provide a broadened perspective on the impact of Aβ on the cause and outcomes of PSCI in clinical practice. Identifying amyloid pathology in patients with PSCI will help select patients who need more focused treatments such as acetylcholinesterase inhibitors

**Trial registration:**

Clinical Research Information Service identifier: KCT0005086

## 1 Introduction

Poststroke cognitive impairment (PSCI) is common, occurs within 3 months after a stroke (or even later), and is associated with poor outcomes.^[1,2]^ The combination of neuroanatomical lesions caused by stroke, other white-matter lesions, and cerebral microbleeds due to small-vessel diseases, and Alzheimer disease (AD) pathologies are known to contribute to PSCI,^[2]^ but our understanding of the role of amyloid pathology in PSCI is still insufficient. Considering a recent comprehensive review,^[3]^ impaired perivascular-space integrity, inflammation, hypoxia, and blood–brain barrier breakdown after stroke can all lead to accelerated deposition of β-amyloid (Aβ) within brain parenchyma and cerebral vessel walls and exacerbations of cerebral amyloid angiopathies. This Aβ deposition in the brain parenchyma would then be the initiating event that leads to synaptic dysfunction and the induction of both cognitive decline and dementia. Despite these proposed Aβ mechanisms, study results for the impact of Aβ on PSCI have been mixed. Several previous reports have supported faster cognitive decline with amyloid pathology in PSCI.^[4–7]^ The results of Mao et al^[7]^ which considered thyroid function in addition to Aβ, indicated positive correlations between Aβ and free thyroxin with PSCI progression, suggesting that these indicators have the potential to predict disease progression and outcome. A 3-year longitudinal study that examined cerebral infarction, hemorrhage, and transient ischemic attack (TIA) reported rapid PSCI progression in amyloid-positive patients.^[4]^ On the other hand, amyloid-pathology prevalence in PSCI was found not to increase in cognitively healthy stroke survivors, suggesting that factors other than amyloid pathology were likely contributors to the development of PSCI.^[8]^ Moreover, Hagberg et al^[9]^ reported that amyloid binding was uncommon and that amyloid deposition did not correlate with cognition in a 7-year longitudinal study. Therefore, this study was designed to examine how cognitive-impairment progression in PSCI is related to amyloid pathology. We will investigate amyloid pathology in patients with PSCI, and compare how differently their cognition progress according to the amyloid pathology.

## 2 Methods

### 2.1 Study design

**Figure 1:**
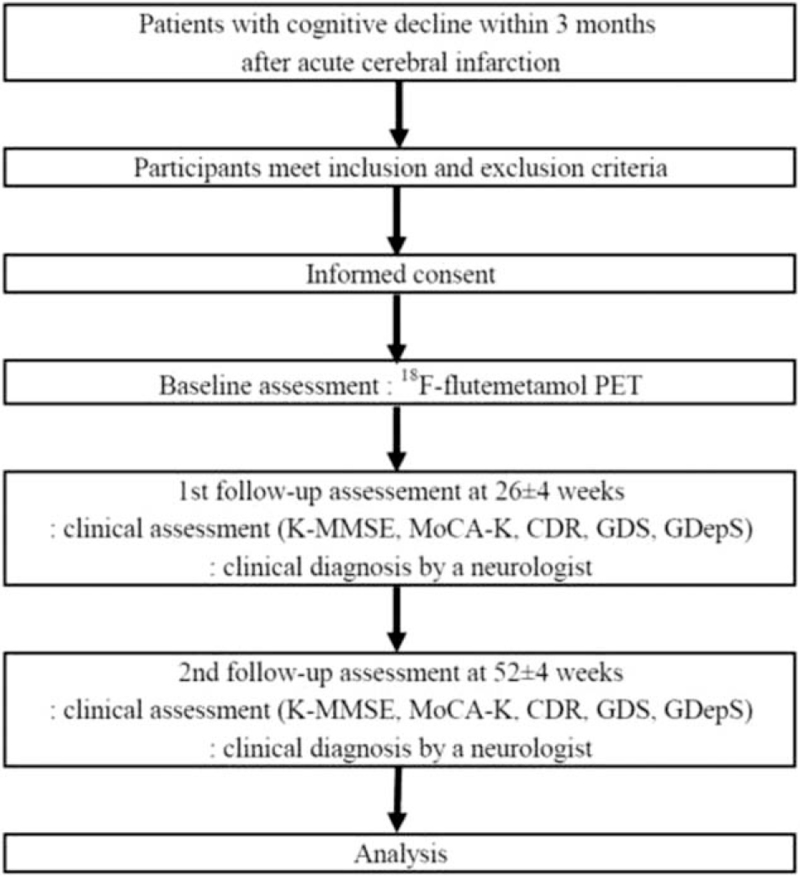
Study flow diagram. CDR = clinical dementia rating, GDepS = geriatric depression scale-short form, GDS = global deterioration scale, K-MMSE = Korean version mini-mental state examination, MoCA-K = Korean version Montreal cognitive assessment, PET = positron emission tomography.

This cognitive decline according to amyloid uptake in patients with PSCI study is designed to be a prospective, multicenter, observational, and hospital-based cohort study using patients suffering from cognitive impairment (CI) after acute stroke. Participants diagnosed with either amnestic mild cognitive impairment (MCI)^[10]^ or mild dementia compatible with Diagnostic and Statistical Manual of Mental Disorders-fourth edition criteria^[11]^ after acute cerebral infarction will be consecutively recruited through 11 hospital neurology clinics. The flow chart of the study was shown in Figure 1.

### 2.2 Study registration

This study trial has been registered on the Clinical Research Information Service (http://cris.nih.go.kr) with an ID of No. KCT0005086).

### 2.3 Participants

Participants diagnosed with either amnestic MCI^[10]^ or mild dementia compatible with Diagnostic and Statistical Manual of Mental Disorders-fourth edition criteria^[11]^ after acute cerebral infarction will be consecutively recruited. The participant inclusion criteria will be: age between 60 and 85 years; complaint of newly developed CI or rapidly aggravated CI within 3 months after an acute cerebral infarction; delayed verbal–memory recall scores using either the Seoul Neuropsychological Screening Battery II^[12]^ or the Literacy Independent Cognitive Assessment^[13]^ are below either −1.0 standard deviation or the 16th percentile; and a clinical dementia rating (CDR)^[14]^ score of 0.5 or 1. We will exclude participants diagnosed with dementia previous to the acute infarction; prescribed acetylcholinesterase inhibitors (AChEIs); diagnosed with strategic infarcts or large cortical infarcts; with other neurological, psychological, or metabolic disorders affecting cognition; and with modified Rankin scale^[15]^ scores >3. See Table 1 for details of these inclusion and exclusion criteria.

**Table 1:** Inclusion and exclusion criteria.

Inclusion criteria
(1) Age between 60 and 85 years
(2) Complaint of newly developed CI or rapidly aggravated CI within 3 months after acute cerebral infarction
(3) Delayed-recall scores of verbal memory in SNSB II or LICA were below −1.0 SD or 16th percentile
(4) CDR was 0.5 or 1

Exclusion criteria
(1) Already had dementia before the acute cerebral infarction
(2) Were prescribed an AChEI before the acute cerebral infarction
(3) Were diagnosed with a strategic infarction or a large cortical infarction
(4) Any other neurological, psychological, or metabolic disorders affecting cognition
(5) mRS >3
(6) Any hearing, visual, or severe language impairments (including aphasia) that could prevent efficient evaluation of the patient
(7) Chronic liver disease, or renal disease being considered for dialysis
(8) Any malignancy not confirmed as cured
(9) Schizophrenia or bipolar disorder based on DSM-IV axis I
(10) Mental retardation
(11) History of encephalitis
(12) Any head trauma that could cause loss of conscientiousness at least 1 hour
(13) Any history of cerebral hemorrhage or brain tumor
(14) Abnormal results of thyroid function test
(15) Cerebral dysfunction related to vitamin B12 or folate deficiency
(16) Neurosyphilis
(17) Had a history of metabolic encephalopathy

AChEI = acetylcholinesterase inhibitor, CDR = clinical dementia rating, CI = cognitive impairment, DSM-IV = Diagnostic and Statistical Manual of Mental Disorders-fourth edition, LICA = Literacy Independent Cognitive Assessment, mRS = modified Rankin scale, SD = standard deviation, SNSB=Seoul Neuropsychological Screening Battery.

### 2.4 Recruitment

We will recruit 196 patients with CI after acute cerebral infarction. The researcher will explain the aim and the details of this study and will obtain informed consent from potential participants before the collection of information. Participation in this study will not affect their clinical treatment.

### 2.5 Intervention

This is an observational study. No study-related interventions are planned. For MCI patients, regardless of amyloid positivity, they will not be treated with AChEIs, whereas only A+ dementia patients will be treated with AChEIs.

### 2.6 Data collection

#### 2.6.1 Assessment measures

For each participant, we will collect basic demographic data (eg, age, sex, years of education), comorbid-disease data that includes vascular risk factors (eg, hypertension, diabetes mellitus, ischemic heart disease, atrial fibrillation, hyperlipidemia, smoking, and alcohol use), medication history, body mass index, and family history of dementia or stroke. All participants will have 3.0-T magnetic resonance imaging (MRI) performed within 1 week of their acute cerebral infarction at the site where it was originally treated. Laboratory tests, including apolipoprotein E genotyping, will be performed before the baseline studies. MRI and MR angiographic imaging resulting from the acute cerebral infarction event will be acquired from each hospital where participants were treated. Included images will be from axial T1-weighted, T2-weighted, T2-weighted fluid-attenuated inversion recovery, diffusion-weighted, gradient-recalled echo, susceptibility-weighted, and coronal T1 sequence imaging, as well as images of intracerebral and extracerebral vessels. We will investigate the location and size of each acute cerebral infarction. We will also evaluate the degree/level of white-matter hyperintensities, the number of lacunes, the number of microbleeds, and the grade of medial-temporal atrophy. For evaluation discrepancies, these will be resolved by investigator consensus. Patient stroke-severity will be assessed at admission using the National Institute of Health Stroke Scale,^[16]^ and the modified Rankin scale. For each patient, we will additionally determine a Hachinski ischemic score^[17]^ and a Trial of ORG 10172 in Acute Stroke Treatment classification^[18]^ for stroke workup.

We will use the informant questionnaire on the cognitive decline in the elderly (IQCODE)^[19]^ to compare a patient's performance after acute infarction with that before the infarction using the information provided by a patient's caregiver. To evaluate participant cognition, we will perform baseline assessments using the Korean Mini-Mental State Examination (K-MMSE),^[20]^ the Korean version of the Montreal Cognitive Assessment (MoCA-K),^[21]^ the CDR, the Global Deterioration Scale,^[22]^ the Korean Instrumental Activities of Daily Living,^[23]^ the Geriatric Depression Scale-short form,^[24]^ and the Literacy Independent Cognitive Assessments or the Seoul Neuropsychological Screening Battery assessments.

The K-MMSE, MoCA-K, CDR, Global Deterioration Scale, and Geriatric Depression Scale-short form will be repeated at 6 and 12 months as follow-up assessments.

#### 2.6.2 Brain positron emission tomography (PET) examination & imaging acquisition

All participants will undergo ^18^F-flutemetamol (FTM) positron emission tomography (PET) at baseline and be observed for 1 year regardless of the results in FTM PET. They will receive an intravenous injection of approximately 185 MBq ^18^F-FTM as a bolus, with the PET scan performed approximately 1 hour postinjection. Each participant's amyloid images will be reviewed first by a nuclear medicine physician at each imaging site and visually rated as either Aβ negative (A−) or positive (A+). Then, all amyloid imaging will be reviewed by a second nuclear-medicine physician, blinded to any clinical information, and categorized visually as either A− or A+. To confirm inter-rater reliability, we will compare the imaging-site results with those of the blinded reviews. In cases of discrepancies between classifications, they will be resolved through consultation with an additional expert.

#### 2.6.3 Data collection methods and management

All paper consents will be secured in the locked cabinet. All the clinical data will be extracted from electronic medical records and checked. Data will be processed de-identified and will be recorded on standardized electronic case report forms.

### 2.7 Study outcomes

The primary outcome measure will be to compare K-MMSE assessment changes (from baseline to week 52) between groups A+ and A− (determined by the visual rating of amyloid PET). The secondary outcome measures will be to compare the A+ and A− groups for differences in the conversion rate to dementia and for changes in MoCA-K assessments (from baseline to week 52). In addition to the clinical factors related to progression, we will investigate the various factors related to amyloid positivity including the IQCODE, as well.

### 2.8 Sample-size estimates

We assumed that a 2-point difference in K-MMSE assessment scores (the primary outcome measure between the A+ and A− groups) after 12 months would be significant. With an alpha value set at 0.05, a beta value of 0.8 (80% power), and a standard deviation value of 5 for change, a total of 78 participants were required for each study group. Assuming a discontinuation rate of 20% during the 12-month study, the total calculated sample size required for the study was 196 participants.

### 2.9 Statistical analysis

We will either apply independent *t* tests for normally distributed continuous variables, the Mann–Whiney *U* test for non-normally distributed variables, or a chi-squared test for categorical variables to compare the 2 participant groups. Linear mixed models will be used to compare any changes in K-MMSE and MoCA-K scores between the A− and A+ groups with age, sex, and education as covariates. A chi-squared test will be used to compare conversion-to-dementia prevalence between the A− and A+ MCI groups, but Fisher exact test will be used if >20% of the values are <5.

### 2.10 Ethics and dissemination

This investigator-initiated clinical trial received funding support from Eisai Korea, but this sponsor was neither involved in the study design nor its proposed operations. The research proposal was approved by the Institutional Review Boards at each of the participating institutions (IRB file No. XC20OIDI0047 at Eunpyeng St. Mary's Hospital), written informed consent will be obtained for all study participants, and all data are to be patient de-identified. The results will be disseminated by publication as a journal article.

## 3 Discussion

Although PSCI occurs frequently in patients with stroke, its prevalence and underlying mechanisms have not been studied in detail. In addition, as a variety of factors are known to contribute to PSCI, it is unclear if Aβ is a predictor of its progression or poststroke dementia, even though Aβ positivity is known to be a strong predictor of progression or conversion to AD from MCI.^[25–27]^ Previous studies of the prevalence of Aβ in PSCI and its effect on the prognosis of PSCI were limited because patients were enrolled even though they underwent initial neuropsychological testing 3 to 6 months after their stroke events, patients with pre-existing dementia were not clearly excluded, cohorts were small, patient assessments were made using computerized tomography results without MRI, a variety of stroke etiologies were used (eg, TIA, hemorrhage, and infarction), and over half of the studies used Pittsburgh compound B as an investigative amyloid tracer, which was not approved for clinical practice.^[4,8,9,28,29]^ Because of these limitations, there is no consensus yet regarding the contribution of Aβ to PSCI and the impact of Aβ on the PSCI prognosis. We hypothesized that Aβ deposition may accelerate neuroinflammation related to stroke, which in turn could result in rapid PSCI progression. With this in mind, we aimed to determine if Aβ affects PSCI cognitive decline. Similar to other studies, the present design also has a limited number of participants and a limited observation period, but the present proposed study overcomes other limitations. In particular, we have tried to register more homogenous groups to find out whether the pathology of AD could contribute to cognition after cerebral infarction. Its strengths are the enrollment of PSCI participants only with acute cerebral infarction (no cerebral hemorrhage or TIA considering that these have different mechanisms and clinical outcomes compared to infarction), the exclusion of strategic infarction or large cortical infarction where the lesions themselves would play a too critical role in cognitive functions or show too much lesion-specific CI, the enrollment of participants who have neuropsychological test results within 3 months of acute infarction, the acquisition and analysis of 3.0-T MRI data, and the use of ^18^F-FTM as the amyloid tracer which is approved for clinical practice.

In conclusion, the present protocol is hypothesis-driven and supported by previous studies on the impact of Aβ on PSCI. If we observe the expected effects, then it will support a link between faster cognitive decline and amyloid pathology and vascular events. The results of this study will likely provide valuable insight for predicting cognitive decline following acute cerebral infarction based on Aβ positivity, or possibly the IQCODE, and for setting AChEI-use standards in PSCI. The goal of this study is to increase our understanding of the long-term outcomes of PSCI in clinical practice.

## Acknowledgments

None

## Author contributions

Yoon B: Investigation, Data curation, Methodology, Writing of original draft; Yang DW, Hong YJ, Kim T, Noh SM, Ku BD, Yang YS, Choi H, Jang JW, Kim S, Kim Y: Investigation, Data curation, Methodology, Review & editing; Park HL: Data curation, Methodology, Review & editing; Shim YS: Conceptualization, Funding acquisition, Investigation, Data curation, Methodology, Review & editing.

**Conceptualization:** YongSoo Shim.

**Data curation:** Bora Yoon, Dong Won Yang, Yun-Jeong Hong, Taewon Kim, Seunghee Na, Sang-Mi Noh, Hye Lim Park, Bon D. Ku, Young Soon Yang, Hojin Choi, Jae-Won Jang, Seongheon Kim, Yerim Kim, YongSoo Shim.

**Funding acquisition:** YongSoo Shim.

**Investigation:** Bora Yoon, Dong Won Yang, Yun-Jeong Hong, Taewon Kim, Seunghee Na, Sang-Mi Noh, Bon D. Ku, Young Soon Yang, Hojin Choi, Jae-Won Jang, Seongheon Kim, Yerim Kim, YongSoo Shim.

**Methodology:** Bora Yoon, Dong Won Yang, Yun-Jeong Hong, Taewon Kim, Seunghee Na, Sang-Mi Noh, Hye Lim Park, Bon D. Ku, Young Soon Yang, Hojin Choi, Jae-Won Jang, Seongheon Kim, Yerim Kim, YongSoo Shim.

**Writing – original draft:** Bora Yoon.

**Writing – review & editing:** Dong Won Yang, Yun-Jeong Hong, Taewon Kim, Seunghee Na, Sang-Mi Noh, Hye Lim Park, Bon D. Ku, Young Soon Yang, Hojin Choi, Jae-Won Jang, Seongheon Kim, Yerim Kim, YongSoo Shim.
